# More is not always better: peat moss mixtures slightly enhance peatland stability

**DOI:** 10.1098/rspb.2023.2622

**Published:** 2024-01-10

**Authors:** Bjorn J. M. Robroek, Giulia Devilee, Yvet Telgenkamp, Carina Härlin, Magdalena N. Steele, Janna M. Barel, Leon P. M. Lamers

**Affiliations:** ^1^ Department of Ecology, Radboud Institute for Biological and Environmental Sciences, Faculty of Science, Radboud University Nijmegen, 6525 AJ Nijmegen, The Netherlands; ^2^ School of Biological Sciences, Faculty of Environmental and Life Sciences, University of Southampton, Southampton, SO17 1BJ, UK; ^3^ Department of Ecosystem and Landscape Dynamics, Institute of Biodiversity and Ecosystem Dynamics (IBED-ELD), University of Amsterdam, P.O. Box 94240, 1090 GE Amsterdam, The Netherlands; ^4^ Länsstyrelsen i Jönköpings län, Store Mosse Nationalpark, 335 74 Hillerstorp, Sweden

**Keywords:** biodiversity-ecosystem functioning, ecological resilience, recovery, replacement series, resistance, *Sphagnum* mosses

## Abstract

Terrestrial wetland ecosystems challenge biodiversity–ecosystem function theory, which generally links high species diversity to stable ecosystem functions. An open question in ecosystem ecology is whether assemblages of co-occurring peat mosses contribute to the stability of peatland ecosystem processes. We conducted a two-species (*Sphagnum cuspidatum*, *Sphagnum medium*) replacement series mesocosm experiment to evaluate the resistance, resilience, and recovery rates of net ecosystem CO_2_ exchange (NEE) under mild and deep water table drawdown. Our results show a positive effect of mild water table drawdown on NEE with no apparent role for peat moss mixture. Our study indicates that the carbon uptake capacity by peat moss mixtures is rather resilient to mild water table drawdown, but seriously affected by deeper drought conditions. Co-occurring peat moss species seem to enhance the resilience of the carbon uptake function (i.e. ability of NEE to return to pre-perturbation levels) of peat moss mixtures only slightly. These findings suggest that assemblages of co-occurring *Sphagnum* mosses do only marginally contribute to the stability of ecosystem functions in peatlands under drought conditions. Above all, our results highlight that predicted severe droughts can gravely affect the sink capacity of peatlands, with only a small extenuating role for peat moss mixtures.

## 1. Introduction

With recent advancements in biodiversity research, evidence is mounting that species rich ecosystems are more stable against environmental pressures [1–3]. The backbone mechanism that underlies increased ecosystem stability within species-rich communities has been suggested to be asynchronous responses of species to changes in climatic and/or environmental conditions [2–4]. Different species within an ecosystem have their own unique adaptations and responses to changing environmental conditions. Essentially, when responses to changing conditions are specific to some extent, the ensuing complementarity enhances the ability of ecosystems to withstand and adapt to environmental stress. Hence, complementarity effects could provide a safety net for ecosystems that facilitates ecosystem stability, and thereby ecosystem functioning, to enviro-climatic pressures.

For terrestrial wetland ecosystems such as peatlands the most important ecosystem function is the ecosystems' ability to sequester and store carbon. Throughout the present interglacial period, northern peatlands have locked up at least 500 Gt of C in the form of peat [5]. Hence, northern peatlands, while they only cover approximately 3% of the land surface [6], are the world's densest C stores [7]. The rapidly changing climate, however, increasingly causes shifts in the C-sink function of peatlands [8–10], and whether these ecosystems can maintain their role as C sinks in the future depends on the interplay between enviro-climatic conditions and processes related to C dynamics. Interestingly, peatlands are species poor in comparison to other terrestrial ecosystems [11], and according to ecological theory on biodiversity-ecosystem functions and stability relationships these systems should be vulnerable to species loss. One pivotal aspect about peatland ecosystems, however, is the presence of a microtopography of hollow-lawn-hummock structures that are characterized by subtle changes in the dominant species present in the plant community. Earlier research has pointed out that the response of these different, but co-occurring, species to alterations in environmental conditions are divergent [12]. At the community level, such asynchronous responses can lead to complementarity between co-occurring species which may contribute to the temporal stability of ecosystem functioning under changing enviro-climatic conditions.

In terrestrial wetland ecosystems such as peatlands, carbon sequestration dynamics largely hinge on the ratio between primary production by aboveground vegetation and decomposition of organic matter by belowground biotic communities. In undisturbed peatlands, the balance between these processes results in net C storage. Crucial therein are hydrological conditions; notably, wet, anoxic conditions hamper decomposition. Moreover, drier conditions and especially droughts impair the ability of the vegetation to sequester carbon [13–15], although compositional changes in the vegetation may mitigate these negative effects on the longer term [16–18]. The stability of the peatland C-sink function is vulnerable to the increasing risk of severe water table drawdown events, especially given current climate predictions of drier and warmer summers. Earlier research on the role of vascular plant community composition on the robustness of peatland carbon dynamics in a changing climate remain inconclusive. Kuiper *et al*., [13] subjected mesocosms with a divergent vascular plant community to a prolonged drought to assess the role of these communities on net carbon uptake. As no clear patterns were found, these authors concluded that the response of peatland plant communities could be largely orchestrated by the peat moss community. Building on that idea, Jassey & Signarbieux [16] suggest that the divergent response in photosynthetic capacity by two *Sphagnum* species to hydrological change can stabilize whole ecosystem carbon dynamics in a changing environment. No studies, hitherto specifically assess the role of *Sphagnum* co-occurrence on the ability of the peat moss assemblage to maintain its function as a carbon sink.

Here, we test a central but unresolved question in terrestrial wetland ecology: whether species mixtures of naturally co-occurring *Sphagnum* moss species—the key ecosystem engineer in many peatlands—enhance the stability of ecosystem functions to environmental perturbation. We used a replacement series experiment to establish the role of peat moss (*Sphagnum*) co-occurrence on net CO_2_ uptake during and after mild and severe drawdown of the water table. We expected the resistance to, and resilience after, drought to be highest in evenly composed mixtures, with largest effects when water tables were deepest.

## 2. Materials and methods

### (a) Sample collection and acclimation

Early September 2021, we collected 40 cores (∅ 22.5 cm, 15 cm depth) of acrotelm peat moss mixtures from a *Sphagnum*-dominated peatland in the Store Mosse National Park, Sweden (57°17′54 N, 14°00′39 E). The cores were gathered to create a single-density replacement series [19] involving two co-occurring *Sphagnum* mosses: *S. cuspidatum* Ehrh. ex Hoffm and *S. medium* Limpr*.* These species overlap in their habitat to some degree but differ in their ecological optimum [12,20]. Together, *S. cuspidatum* and *S. medium* make up greater than 70% (cover) of the field layer in the field site, notably in wet lawn microhabitat. True hummock species (e.g. *S. fuscum*) are rare in the Store Mosse peatland (B.J.M.R. 2010–2023, personal observation). Our sampling strategy resulted in cores with either pure stands (100% cover) of *S. cuspidatum* or *S. medium*, and three species mixtures (75%/25%, 50%/50%, 25%/75%; *n* = 8), with final cover ratios potentially differing by ±5%. All cores were collected from naturally occurring species mixtures; water tables in the field were close (±1 cm) to the peat moss surface. Vascular plants were removed.

The cores, set in tight-fitting PVC buckets (∅ 22.5 cm, 20 cm depth) that contained a 3 cm layer of white sand, were transported to the research facilities of the Radboud Institute for Biological and Environmental Sciences. There, they were acclimatized in a climate room for two weeks, watered with artificial—low nutrient—rainwater [13,14,21] to reach a water level of 1 cm below the *Sphagnum* surface, and then placed in a water bath (12°C, cooled by a Thermo Scientific ThermoFlex1400) for another four weeks of conditioning.

The climate room temperature was maintained at 22°C/15°C. The light regime consisted of a 16 h/8 h day/night cycle and provided 280–290 µmol PAR m^−2^ s^−1^ (Philips GreenPower LED toplighting DR/W/FR_2 400 V). To ensure uniform conditions, the mesocosms were randomly repositioned in the water bath after every greenhouse gas measurement cycle (see below) throughout the experiment.

### (b) Experimental set-up

**Figure 1.**
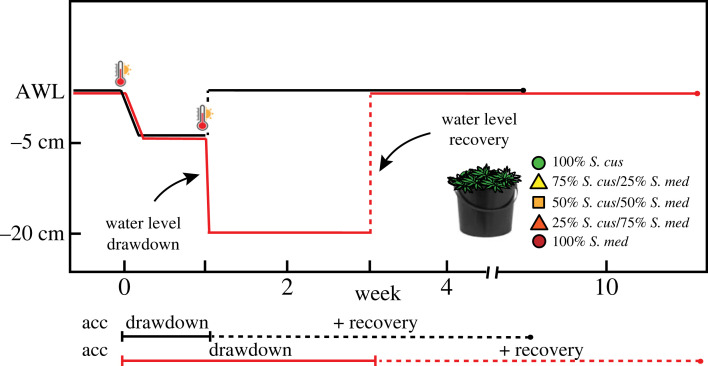
Schematic overview of the experimental water table drawdown treatments. The mild (black line, *n* = 4) and deep drawdown (red line, *n* = 4) for all five *Sphagnum* mixtures (100% *S. cuspidatum*; 75% *S. cuspidatum* / 25% *S. medium*; 50% *S. cuspidatum* / 50% *S. medium*; 25% *S. cuspidatum* / 75% *S. medium*; 100% *S. medium*) were preceded by an acclimation period (acc) in which the water levels were kept constant (AWL, ambient water level; −1 cm). Water table draw-down was initiated by a warming event (indicated by the thermometer pictogram). After a no-precipitation period of one (mild drawdown) or three (deep drawdown) weeks, water levels were brought back to AWL after which the mesocosms were left to recover for eight weeks.

Following the acclimation period, the eight mesocosms for the five compositionally distinct *Sphagnum* communities were randomly assigned an experimental treatment (*n* = 4): mild drawdown—consisting of a water table drawdown of 5 cm followed by a seven-day drought period in which no precipitation was added—or deep drawdown—where the initial seven-day mild drawdown was followed by a water table drawdown of 20 cm and an additional two-week without precipitation. Every drawdown event was preceded by mimicking a heatwave, which under natural conditions precedes a drop in water table. To keep the number of heat events even among all mesocosms, we also applied a heatwave preceding the water table recovery after the mild drawdown event. The heatwaves were realized by increasing the climate room temperature to approximately 35°C for 10 hrs. using twelve infrared lamps (Philips IR 250 RH IR2 230–250 V 250 W) that were installed 70 cm above the surface of the mesocosms. The mesocosm during this period remained embedded in the 12°C water bath environment. Both drought treatments were trailed by a rewetting (= recovery) period, where the water table was raised to pre-drawdown levels using a watering can. The rewetting period lasted eight weeks (figure 1).

### (c) Carbon dioxide gas fluxes

Throughout the experiment, we used an airtight transparent acrylic chamber (∅ 29 cm, height 30 cm) equipped with an internal fan to measure carbon dioxide (CO_2_). The chamber sealed onto the mesocosms and was connected in a closed loop to a LI-7810 CH_4_/CO_2_/H_2_O Trace Gas Analyzer (LI-COR Biosciences). Gas measurements were performed with a frequency of 1 hz over a 120 s interval; the chamber was vented between measurements. Measurements occurred three times—11, 9 and 1 days before water table drawdown—in the acclimation period, four times per week in the water table drawdown period, and two times per week in the recovery period.

Measurements with the transparent chamber represent net ecosystem exchange (NEE). CO_2_ fluxes were calculated using the *R* package *FluxCalR* [22], which makes use of the change in gas concentration in the chamber over time. Due to instability in the gas flux measurements, we used a dead band of 30 s for the NEE calculations. The ecological sign convention was used for the NEE data; hence, positive flux values indicate CO_2_ uptake, while negative flux values indicate CO_2_ loss to the atmosphere.

### (d) Ecosystem stability measures

We calculated ecosystems stability measures for all individual mesocosms based on the framework described by [23]. First, resistance to water table drawdown was calculated for both drawdown treatments as the proximity of net CO_2_ exchange (NEE) at the end of the treatment period to pre-drawdown NEE levels. Resistance was calculated as the dimensionless variable $\Omega \equiv \bar{NEE_{\mathrm{acc}}}/(NEE_{d}-\bar{NEE_{\mathrm{acc}}})$, where $\bar{NEE_{\mathrm{acc}}}$ is the mean net CO_2_ exchange during the acclimation period, and $NEE_{d}$ is the net CO_2_ exchange at the last day of the water table drawdown treatment. As the effect of water table drawdown on NEE can be bidirectional, opposite to Isbell *et al*. [23], we used the true values for Ω. As such, resistance values become asymmetric; reductions in NEE as a response to water table drawdown in comparison to NEE levels in the acclimation period ($NEE_{\mathrm{acc}}$) result in negative values of Ω with Ω=−2 indicating NEE levels to half, while increased NEE values as a response to drawdown result in positive values, with Ω=1 indicating a doubling in NEE. Values of Ω≅0 indicate a small resistance; large Ω values (positive and negative) indicate high resistance. Second, resilience indicates the ability of a perturbed process to return to pre-perturbation levels, and is expressed as the dimensionless variable $\Delta \equiv |(NEE_{d}-\bar{NEE_{\mathrm{acc}}})/(NEE_{\mathrm{rec}}-\bar{NEE_{\mathrm{acc}}})|$, where $\bar{NEE_{\mathrm{acc}}}$ is the mean net CO_2_ exchange during the acclimation period, $NEE_{d}$ is the net CO_2_ exchange at the last day of the water table drawdown treatment, and $NEE_{\mathrm{rec}}$ is the net CO_2_ exchange at the last day of the recovery period. High values of Δ indicate a high capacity of the ecosystems to return to pre-drawdown process levels.

### (e) Data analyses

Prior to any (statistical) analysis, one mesocosm with a 75/25% *S. cuspidatum*/*S. medium* cover and assigned to the deep drawdown treatment (DD_75/25) was omitted from the dataset, due to a leaking container during the experiment.

We used linear mixed-effects models to test whether drawdown treatment and *Sphagnum* mixture affected net ecosystem CO_2_ exchange (NEE), while taking the repeated measure structure of the data into account. Repeated measures models were fitted within the experimental periods (i.e. the acclimation, the mild drawdown, the deep drawdown and the recovery period) separately. All interactions between the fixed effects treatment (drawdown treatment) and *Sphagnum* mixture (100% *S. cuspidatum*; 75% *S. cuspidatum* / 25% *S. medium*; 50% *S. cuspidatum* / 50% *S. medium*; 25% *S. cuspidatum* / 75% *S. medium;* 100% *S. medium)* and the repeated factor (day; within experimental period) were accounted for. For the acclimation and the mild drawdown periods, all mesocosm (*n* = 8, total; i.e. those assigned to the mild and to the deep drawdown treatment) were considered in the analyses, as during these periods the hydrological regimens where identical for both treatments. The effect of deep drawdown was then assessed on the specifically assigned mesocosms only (*n* = 4). The recovery periods—which started at different times for the two drawdown treatments—were aligned to the start of the recovery period. For the water table drawdown and recovery periods, response slopes were calculated for each individual mesocosm. The effects of community composition on the response-slopes were analysed using analysis of variance.

Whether resistance, resilience and the NEE recovery rates depended on drawdown treatment and *Sphagnum* mixture was tested with linear mixed effects models that included all interactions terms. All analyses were performed in the R statistical environment, using version 4.2.3 (‘Shortstop Beagle’).

## 3. Results

### (a) Pre-drought acclimation

Net ecosystem CO_2_ exchange (NEE) during the acclimation phase varied between 0.74 and 1.71 µmol CO_2_ m^−2^ s^−1^ (electronic supplementary material, figure S1) and did not differ between assigned drawdown treatments (*F*_1,106_ = 0.012, *p* = 0.91), nor between community composition (*F*_4,106_ = 0.199, *p* = 0.94). Repeated measures analysis indicated no changes in NEE during the acclimation period (*F*_1,62_ = 0.391, *p* = 0.53). Indeed, neither the mesocosms assigned to either the mild drawdown or the deep drawdown treatment (*F*_1,62_ = 1.426, *p* = 0.24), nor the different mixtures (*F*_4,62_ = 1.313, *p* = 0.27), responded differently over time (electronic supplementary material, figure S1).

### (b) Mild water table drawdown

**Figure 2.**
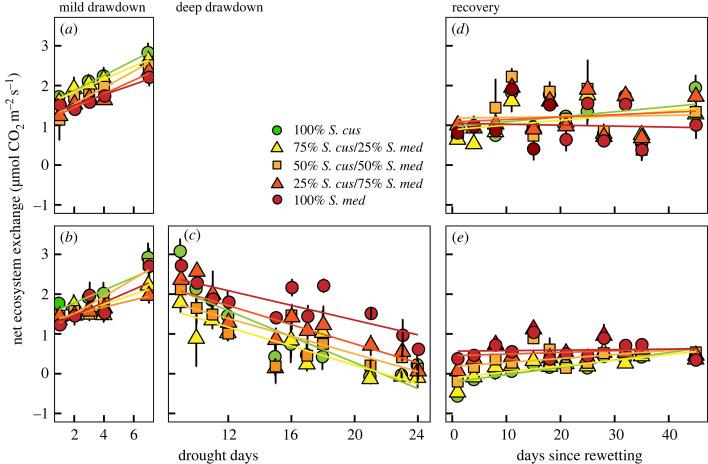
Responses of net ecosystem CO_2_ exchange (NEE) to (*a*,*b*) a mild (*n* = 4) and (*c*) a consecutive deep water table drawdown period (*n* = 4). Following these mild and deep drawdown events, (*d*,*e*) all mesocosms were rewetted for 40 days (*n* = 4). Responses in NEE are shown for the five *Sphagnum* mixtures: 100% *S. cuspidatum*; 75% *S. cuspidatum* / 25% *S. medium*; 50% *S. cuspidatum* / 50% *S. medium*; 25% *S. cuspidatum* / 75% *S. medium*; 100% *S. medium*.

Net ecosystem CO_2_ exchange (NEE) in all treatments increased within the mild drawdown period (*F*_1,145_ = 209.6, *p* < 0.001). Mesocosms assigned to the mild and deep drawdown treatments responded similarly to the initial seven-day mild drawdown (*F*_1,29_ = 0.660, *p* = 0.42). Hence, further analyses on the effects of *Sphagnum* mixture were performed on all eight mesocosms. While mixture seemed to marginally play a role in NEE (*F*_4,34_ = 2.216, *p* = 0.09)—with highest and lowest overall values in the *S. cuspidatum* and *S. medium* monocultures, respectively (figure 2*a,b*)—their response to mild drawdown was similar (*F*_4,150_ = 1.544, *p* = 0.19). The latter was confirmed by comparing the NEE response-slopes of the mild drought mesocosms (table 1). Treatment (*F*_1,28_ = 0.009, *p* = 0.92), nor composition (*F*_4,28_ = 0.824, *p* = 0.52) had an effect on the change of NEE over the mild drought period, and response-slopes for the different mixtures were similar between mesocosms assigned to two drawdown treatments (*F*_4,28_ = 0.703, *p* = 0.60; figure 2*a,b*).

**Table 1.** Response slopes (± st. err) of the net ecosystem CO_2_ exchange (NEE) during the mild water table drawdown (*n* = 8) and the deep drawdown (*n* = 4) period for the five *Sphagnum* mixtures: 100% *S. cuspidatum*; 75% *S. cuspidatum* / 25% *S. medium*; 50% *S. cuspidatum* / 50% *S. medium*; 25% *S. cuspidatum* / 75% *S. medium*; 100% *S. medium.*

100% S. cus	75% S. cus /25% S. med	50% S. cus / 50% S. med	25% S. cus / 75% S. med	100% S. med
mild water table drawdown treatment				
0.20 ± 0.03	0.14 ± 0.03	0.21 ± 0.04	0.14 ± 0.03	0.19 ± 0.05
deep water table drawdown treatment				
−0.18 ± 0.02	−0.11 ± 0.02	−0.11 ± 0.02	−0.13 ± 0.04	−0.09 ± 0.01

### (c) Deep water table drawdown

Immediately after the onset of the deep drawdown treatment, net CO_2_ exchange (NEE) started to decrease in all *Sphagnum* mixtures (*F*_1,184_ = 218.2, *p* ≤ 0.001; figure 2*c*). NEE in the different mixtures was different (*F*_4,14_ = 5.100, *p* = 0.010), and *Sphagnum* mixtures with different composition exhibited different response rates to deep water table drawdown (*F*_4,184_ = 3.270, *p* = 0.013). Indeed, while non-significant, slopes (i.e. response rates) were highest for pure *S. cuspidatum* and lowest for pure *S. medium* (*F*_4,13_ = 2.320, *p* = 0.112; table 1; figure 2*c*). Pairwise comparison of the response rates of the different mixtures underpinned these finding; the decline in NEE during deep drawdown was marginally faster for *S. cuspidatum* than for *S. medium* (Tukey’ *t* = −2.676, *p* = 0.113). Additional pairwise comparisons revealed no differences between *Sphagnum* mixtures.

### (d) Recovery after water table drawdown

Rewetting of the mesocosms resulted in an immediate decrease in NEE for the mesocosms that had previously been subjected to a mild water table drawdown (figure 2*d*), while such decrease was less obvious (even absent) for the mesocosms previously subjected to a more subsequent deeper drawdown event (figure 2*e*). NEE in the mild drawdown mesocosms during the recovery period only changed marginally over time (*F*_1,213_ = 3.213, *p* = 0.074; figure 2*d*), which was consistent for the different compositions. The mesocosms previously subjected to the deep drawdown responded more observably to rewetting (*F*_1,202_ = 55.05, *p* ≤ 0.001; figure 2*e*), yet the effect of rewetting was dependent on *Sphagnum* mixture (*F*_4,202_ = 5.499, *p* ≤ 0.001; figure 2*e*), with faster recovery for mesocosms with a ≥75% cover of *S. cuspidatum*.

### (e) Resistance, resilience, and recovery rates

**Figure 3.**
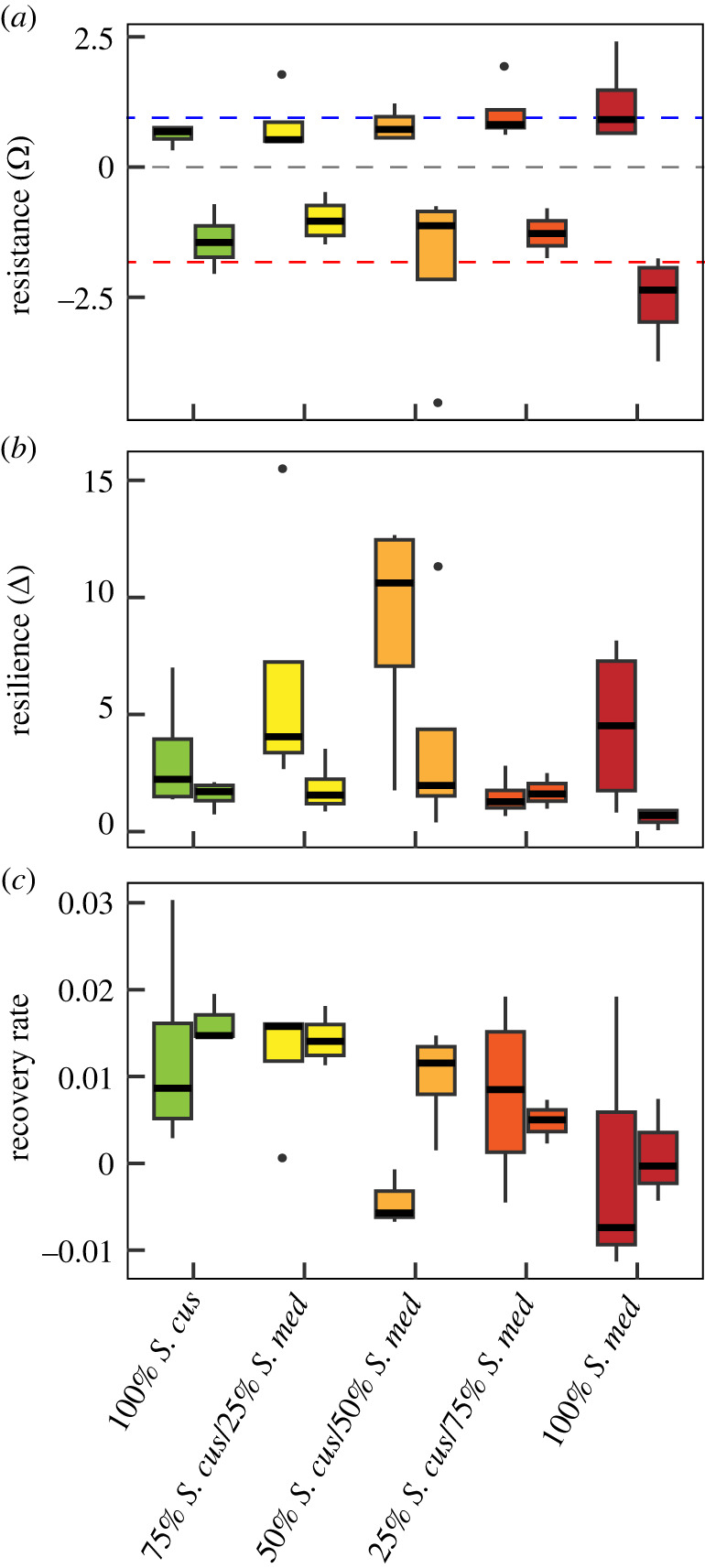
(*a*) Resistance (*Ω*), (*b*) resilience (*Δ*), and (*c*) recovery rate for the mild (left boxplot; *n* = 4) and deep (right boxplot; *n* = 4) water table drawdown treatment for the five *Sphagnum* mixtures: 100% *S. cuspidatum*; 75% *S. cuspidatum* / 25% *S. medium*; 50% *S. cuspidatum* / 50% *S. medium*; 25% *S. cuspidatum* / 75% *S. medium*; 100% *S. medium*. The blue dashed line in a. indicates a doubling in NEE during drawdown, while the red dashed line indicates halved NEE values due to drought.

Resistance to drying was almost similar, though opposite, for the mesocosms subjected to mild and severe drawdown events. Mild drawdown almost doubled NEE values, while severe drawdown halved NEE values. Most interesting in the context of our study, resistance was not affected by *Sphagnum* mixture (*F*_4,29_ = 0.762, *p* = 0.559; figure 3*a*). Resilience to drying was highest for the mesocosms subjected to mild water table drawdown (*F*_1,29_ = 7.690, *p* ≤ 0.001; figure 3*b*). Hence, resistance and resilience were both hampered by deep water table drawdown. Resilience to drying was mixture dependent (*F*_4,29_ = 2.659, *p* ≤ 0.05), and notably after mild drawdown appeared to be enhanced in the *Sphagnum* communities with a 1-to-1 ratio in *S. cuspidatum* and *S. medium* (figure 3*b*). Rates of recovery (i.e. the slopes of NEE over the recovery period) differed between *Sphagnum* mixtures for those mesocosms that were previously subjected to deep drawdown (*F*_4,12_ = 6.782, *p* = 0.004), but not for those previously subjected to mild drawdown (*F*_4,13_ = 1.598, *p* = 0.234; figure 3*c*).

## 4. Discussion

Climate models indicate increased occurrences of warm and dry summers [24], especially for Europe [25]. Indeed, the Copernicus Climate Change Service in its European State of the Climate 2022 report [26] highlighted that the last eight years have been the warmest on record, globally. Further, Europe has experienced exceptional drought stress, with many implications to natural ecosystems. Assessing the effects of drought-induced water table drawdown in peatlands is needed as the carbon sink function of these ecosystems largely depend on high water tables and a sufficient supply of rainwater [14]. Here, we experimentally test the role of peat moss species assembly on the C sink function of peatlands under drying conditions.

### (a) Water table drawdown depth matters

Our work reveals a strong divergent response in net ecosystem CO_2_ exchange (NEE) by the peat moss mixtures that were exposed to mild and deep water table drawdown, with the latter indicating long-term consequences on peatland functioning [27]. Mild drawdown caused NEE to increase; a response that was shared and similar for all peat moss community compositions. These results echo findings in a previous study by Robroek *et al*. [12] and can be attributed to the higher CO_2_ diffusion between the atmosphere and the moss tissue at the initial phase of drying [16,28]. Such diffusion effect is supported by our observation that levels of NEE returned to pre-drought levels almost immediately after rewetting. Moreover, ­NEE was slightly below pre-drought levels, likely due to cell damage during the drying-rewetting phase [29–31], but recovered steadily throughout the rewetting phase. This apparent recovery was higher for *S. cuspidatum* than for *S. medium*, resulting in differences in resilience to mild water table drawdown between the peat moss assemblages (see below).

Prolongation and intensification of the drought period by an additional two weeks (i.e. with deep water table drawdown) resulted in a gradual decrease in NEE by the peat moss communities. Interestingly, this decrease seems slightly stronger in *S. cuspidatum* monocultures as compared to *S. medium* monocultures. These results are in line with earlier work which showed such interspecific differences in CO_2_ assimilation responses to drought [12]. Furthermore, our results reflect the ecology of the species in this study. While often growing in proximity with large overlap in hydrological niche, their abilities to resist and persist drought are different. *Sphagnum cuspidatum* is a hollow/lawn dwelling species, tied to a proximate water table. Due to its growth form, *S. cuspidatum* is not able to hold water well [32]. *S. medium* grows denser and is in general better able to hold water (but see Bengtsson *et al*. [32] for an opposite finding). More important, *S. medium* is able to assimilate carbon at lower contents of water as compared to *S. cuspidatum* [32], which likely explains why NEE for the latter species is lower, and declines faster, than for *S. medium*. The effects of deep drawdown seem long lasting with low recovery rates [27,33], resulting in low ecosystem resilience.

### (b) Stability of net ecosystem CO_2_ exchange under water table drawdown

Our findings reiterate earlier work that show the presence of a hydrological threshold that, if passed, causes the ecosystem to shift states [17,34]. Moreover, the divergent response of the two co-occurring species—low resistance but high recovery for *S. cuspidatum*, and high resistance but low recovery for *S. medium*—to mild drawdown seemed to result in overall higher resilience of NEE in co-occurring peat mosses. The overperformance in resilience in mixtures, notably when subjected to mild water table drawdown, suggests complementarity due to abiotic facilitation [4,35].

Our results also suggest a role of *Sphagnum* moss community composition on the effects on ecosystem stability under drying conditions. The hollow/lawn-dwelling *S. cuspidatum*, for example, shows relative low resistance to drying conditions, as compared to the lawn/hummock-dwelling *S. medium*. But, despite their lower resistance against prolonged water table drawdown, pure *S. cuspidatum* stands were remarkably capable of recovering [16]. Nevertheless, CO_2_ fluxes at the end of the experiment, remained far (i.e. approx. 45%) lower than pre-drought values. The lower recovery in pure *S. medium* stands suggests that deep drought lags on species performance; a pattern which has earlier been observed in the Italian Alps after extreme summer drought [27]. These community composition specific differences in stability metrics we observe may on the long run be reflected in changes in the peat moss community [34], with drought tolerant species to outcompete non-tolerant species [36]. The resulting homogenization in the peat moss community may erode the peatland's ability to return to pre-drought carbon sequestration rates as our results indicate that non-conspecific neighbours can help to stabilize peatland carbon uptake.

## 5. Conclusion

The resilience of peatlands to enviro-climatic disturbances remains poorly studied. Recent work has addressed the resilience of restored peatlands, yet the outcomes or restoration efforts are variable. While a certain degree of resilience on the carbon sink function of rewetted degraded peatland has been reported [18,37,38], the time scales of such recovery may much diverge from current needs for peatland management and restoration [39]. Furthermore, resilience can be a local effect and the recovery of peatland biotic communities has been reported to lag behind functional recovery [40]. On the backdrop of this apparent ambiguity, protecting intact peatlands and their associated functions is a matter of urgency [38]. In this light, our work indicates that resilience of the C sink function in intact peatlands, which is underpinned by the C sink function of peat moss community, depends on drought intensity. Much of the sink capacity of the peat moss community can be lost under prolonged drought, with low resilience and slow recovery rates.

## Data Availability

All data and code are available through Dryad [41]. All data that support this manuscript are also available through DANS, the Netherlands institute for permanent access to digital research resources (https://dans.knaw.nl/en): doi:10.17026/dans-xfk-72pn [42]. Supplementary material is available online [43].

## Statement of inclusion

Our study brings together authors from different career stages, including an MSc student, PhD students, an early career researcher and tenured staff, who engaged at multiple stages in the study to ensure a broad perspective on the topic. All input was valued equally and part in the final version of our manuscript. Our study incudes one the managers of the nature reserve where material for our study was taken, which led to critical and valuable stakeholder input to our work.
